# 4-(2-Methyl­piperidin-1-ylcarbon­yl)pyridinium hexachloridoantimonate(V)

**DOI:** 10.1107/S1600536809036587

**Published:** 2009-09-16

**Authors:** Bo Wang

**Affiliations:** a Ordered Matter Science Research Center, Southeast University, Nanjing 210096, People’s Republic of China

## Abstract

In the hexa­chlorido­animonate anion of the title compound, (C_12_H_17_N_2_O)[SbCl_6_], the Sb^5+^ion is in a slightly distorted octa­hedral coordination. In the 4-(2-methyl­piperidine-1-carbon­yl) pyridinium cation, the dihedral angle between the mean planes of the pyridine and piperzine rings is 66.3 (3)°. The mean plane of the carbonyl group is twisted by 80.5 (7)° and 42.7 (4)° relative to the mean planes of the pyridine and piperzine rings, respectively. The methyl group is in an *R* configuration relative to the piperidine ring which is in a slightly distorted chair conformation. The crystal packing is stabilized by N—H⋯O hydrogen bonds between cations, which form infinite zigzag chains parallel to [010].

## Related literature

For the use of halogenidoanti­monate salts in the study of phase transitions in dielectric–ferroelectric materials, see: Jakubas *et al.* (2005 ▶); Bednarska-Bolek *et al.* (2000 ▶). For related structures, see: Chen (2009 ▶); Clemente & Marzotto (2003 ▶); Kulicka *et al.* (2006 ▶). For puckering parameters, see: Cremer & Pople (1975 ▶).
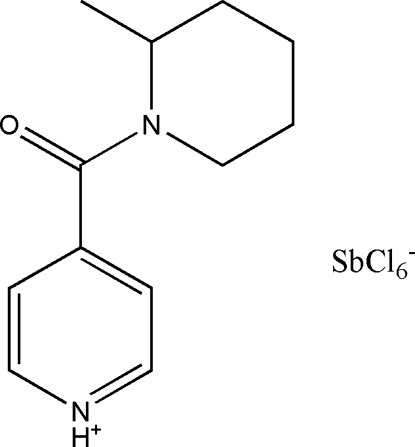


## Experimental

### 

#### Crystal data

(C_12_H_17_N_2_O)[SbCl_6_]
*M* *_r_* = 539.73
Monoclinic, 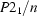

*a* = 8.1067 (16) Å
*b* = 12.700 (3) Å
*c* = 19.677 (4) Å
β = 99.06 (3)°
*V* = 2000.6 (7) Å^3^
*Z* = 4
Mo *K*α radiation
μ = 2.18 mm^−1^
*T* = 298 K
0.20 × 0.20 × 0.20 mm

#### Data collection

Rigaku SCXmini diffractometer
Absorption correction: multi-scan (*CrystalClear*; Rigaku, 2005 ▶) *T*_min_ = 0.638, *T*_max_ = 0.646
17300 measured reflections
3918 independent reflections
2731 reflections with *I* > 2σ(*I*)
*R*_int_ = 0.065

#### Refinement

*R*[*F* ^2^ > 2σ(*F* ^2^)] = 0.072
*wR*(*F* ^2^) = 0.178
*S* = 1.06
3918 reflections
199 parameters
8 restraints
H-atom parameters constrained
Δρ_max_ = 1.08 e Å^−3^
Δρ_min_ = −0.82 e Å^−3^

### 

Data collection: *CrystalClear* (Rigaku 2005 ▶); cell refinement: *CrystalClear*; data reduction: *CrystalClear*; program(s) used to solve structure: *SHELXS97* (Sheldrick, 2008 ▶); program(s) used to refine structure: *SHELXL97* (Sheldrick, 2008 ▶); molecular graphics: *SHELXTL* (Sheldrick, 2008 ▶); software used to prepare material for publication: *PRPKAPPA* (Ferguson, 1999 ▶).

## Supplementary Material

Crystal structure: contains datablocks I, New_Global_Publ_Block. DOI: 10.1107/S1600536809036587/jj2004sup1.cif

Structure factors: contains datablocks I. DOI: 10.1107/S1600536809036587/jj2004Isup2.hkl

Additional supplementary materials:  crystallographic information; 3D view; checkCIF report

## Figures and Tables

**Table 1:** Hydrogen-bond geometry (Å, °)

*D*—H⋯*A*	*D*—H	H⋯*A*	*D*⋯*A*	*D*—H⋯*A*
N1—H1*B*⋯O1^i^	0.86	1.87	2.689 (9)	159

Symmetry code: (i) 
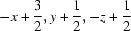
.

**Fig. 1.**
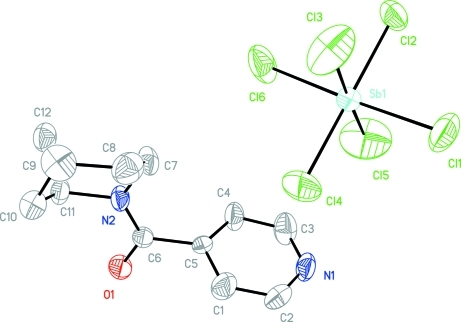
The molecular structure of the title compound, with the atomic numbering scheme. Displacement ellipsoids are drawn at the 30% probability level, and all H atoms have been omitted for clarity.

**Fig. 2.**
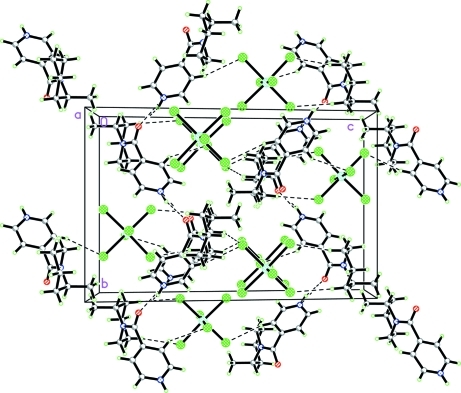
A view of the crystal packing of the title compound, Dashed lines indicate N–H···O hydrogen bonds which form infinite, one-dimensional chains along the (011) plane of the unit cell.

## supplementary crystallographic information

### Comment

Halogenidoantimonate salts are used to study phase transitions in dielectric-ferroelectric materials (Jakubas *et al.*, 2005; Bednarska-Bolek *et al.*, 2000). In support of this work, crystal structures of pyridinium hexachloridoantimonate, (Clemente & Marzotto, 2003),4-aminopyridinium hexachloridoantimonate (Kulicka *et al.*, 2006) and diisonicotinium pentachloridoantimonate monohydrate (Chen, 2009) have beenreported. In continuation of our studies on halogenoantimonate salts, we report the crystal structure of the title compound, C_12_H_17_N2_O_^+^. SbCl_6_^-^, (I).

In the cation (4-(2-methylpiperidine-1-carbonyl) pyridinium), the pyridine N atom is protonated. The piperidine ring (N2/C7—C11) adopts a slightly distorted chair conformation (Cremer & Pople, 1975) with puckering parameters Q, θ and φ of 0.564 (4) Å, 177.0 (6)° and 177.084 (5)°, respectively (Fig. 1). For an ideal chair θ has a value of 0 or 180°. The mean plane of the carbonyl group is twisted relative to the mean planes of the pyridine and piperzine rngs by 80.5 (7)° and 42.7 (4)°, respectively. The dihedral angle between the mean planes of pyridine and piperzine rings is 66.3 (3)°. In the anion the Sb atom is hexacoordinated with Cl atoms in a slightly distorted octahedral conformation. The Sb—Cl bond lengths (2.330 (3) to 2.348 (3) Å) are similar to that observed in pyridinium hexachlorido-antimony(V) (2.32 (1)–2.35 (5) Å; Clemente & Marzotto, 2003) and slightly shorter than that reported for 4-aminopyridinium hexachloridoantimonate (2.3608 (8)–2.3912 (7) Å; Kulicka *et al.*,2006). Crystal packing is stabilized by N1–H1B···O1 hydrogen bonds between cations which form infinite zigzag chains parallel to [010] (Fig. 2).

### Experimental

A mixture of 4-(2-methylpiperidine-1-carbonyl)pyridine(1 mmol), SbCl_5_ (1 mmol), ethanol(8 ml) and a few drops of HCl (6 mol/*L*) was stirred in a beaker. There were many solid powders produced and the solution was filtered. Colorless single crystals of the title compound suitable for X-ray analysis were obtained on slow evaporation of the solvents over a period of 48 h.

### Refinement

Positional parameters of all the H atoms were calculated geometrically and were allowed to ride on the C atoms to which they are bonded, with *U*_iso_(H) = 1.2*U*_eq_(C).

### Crystal data

**Table:**

(C_12_H_17_N_2_O)[SbCl_6_]	*F*(000) = 1056
*M**_r_* = 539.73	*D*_x_ = 1.792 Mg m^−^^3^
Monoclinic, *P*2_1_/*n*	Mo *K*α radiation, λ = 0.71073 Å
Hall symbol: -P 2yn	Cell parameters from 7472 reflections
*a* = 8.1067 (16) Å	θ = 3.0–27.7°
*b* = 12.700 (3) Å	µ = 2.18 mm^−^^1^
*c* = 19.677 (4) Å	*T* = 298 K
β = 99.06 (3)°	Prism, colourless
*V* = 2000.6 (7) Å^3^	0.20 × 0.20 × 0.20 mm
*Z* = 4	

### Data collection

**Table:**

Rigaku SCXmini diffractometer	3918 independent reflections
Radiation source: fine-focus sealed tube	2731 reflections with *I* > 2σ(*I*)
graphite	*R*_int_ = 0.065
Detector resolution: 13.6612 pixels mm^-1^	θ_max_ = 26.0°, θ_min_ = 3.0°
ω scans	*h* = −9→9
Absorption correction: multi-scan (*CrystalClear*; Rigaku, 2005)	*k* = −15→15
*T*_min_ = 0.638, *T*_max_ = 0.646	*l* = −24→24
17300 measured reflections	

### Refinement

**Table:**

Refinement on *F*^2^	Primary atom site location: structure-invariant direct methods
Least-squares matrix: full	Secondary atom site location: difference Fourier map
*R*[*F*^2^ > 2σ(*F*^2^)] = 0.072	Hydrogen site location: inferred from neighbouring sites
*wR*(*F*^2^) = 0.178	H-atom parameters constrained
*S* = 1.06	*w* = 1/[σ^2^(*F*_o_^2^) + (0.0594*P*)^2^ + 10.7201*P*] where *P* = (*F*_o_^2^ + 2*F*_c_^2^)/3
3918 reflections	(Δ/σ)_max_ < 0.001
199 parameters	Δρ_max_ = 1.08 e Å^−^^3^
8 restraints	Δρ_min_ = −0.82 e Å^−^^3^

### Special details

**Table:**

Geometry. All e.s.d.'s (except the e.s.d. in the dihedral angle between two l.s. planes) are estimated using the full covariance matrix. The cell e.s.d.'s are taken into account individually in the estimation of e.s.d.'s in distances, angles and torsion angles; correlations between e.s.d.'s in cell parameters are only used when they are defined by crystal symmetry. An approximate (isotropic) treatment of cell e.s.d.'s is used for estimating e.s.d.'s involving l.s. planes.
Refinement. Refinement of *F*^2^ against ALL reflections. The weighted *R*-factor *wR* and goodness of fit *S* are based on *F*^2^, conventional *R*-factors *R* are based on *F*, with *F* set to zero for negative *F*^2^. The threshold expression of *F*^2^ > σ(*F*^2^) is used only for calculating *R*-factors(gt) *etc*. and is not relevant to the choice of reflections for refinement. *R*-factors based on *F*^2^ are statistically about twice as large as those based on *F*, and *R*- factors based on ALL data will be even larger.

### Fractional atomic coordinates and isotropic or equivalent isotropic displacement parameters (Å^2^)

**Table:**

	*x*	*y*	*z*	*U*_iso_*/*U*_eq_	
C1	0.5950 (13)	0.2859 (8)	0.2451 (5)	0.092 (3)	
H1A	0.5293	0.2434	0.2685	0.110*	
C2	0.6745 (15)	0.3710 (9)	0.2774 (6)	0.107 (4)	
H2A	0.6625	0.3868	0.3226	0.129*	
C3	0.7859 (12)	0.4104 (8)	0.1821 (6)	0.089 (3)	
H3A	0.8534	0.4541	0.1604	0.107*	
C4	0.7082 (13)	0.3263 (7)	0.1474 (5)	0.076 (3)	
H4A	0.7221	0.3128	0.1022	0.091*	
C5	0.6107 (9)	0.2626 (6)	0.1797 (4)	0.0474 (18)	
C6	0.5366 (10)	0.1615 (6)	0.1476 (4)	0.055 (2)	
C7	0.2968 (10)	0.2610 (6)	0.0863 (6)	0.067 (3)	
H7A	0.3572	0.3219	0.1069	0.080*	
H7B	0.2776	0.2704	0.0368	0.080*	
C8	0.1374 (15)	0.2526 (9)	0.1110 (6)	0.094 (3)	
H8A	0.0691	0.3133	0.0955	0.113*	
H8B	0.1566	0.2531	0.1610	0.113*	
C9	0.0415 (15)	0.1501 (9)	0.0851 (7)	0.102 (4)	
H9A	−0.0581	0.1434	0.1064	0.123*	
H9B	0.0074	0.1538	0.0356	0.123*	
C10	0.1563 (13)	0.0526 (8)	0.1035 (5)	0.084 (3)	
H10A	0.1810	0.0446	0.1530	0.100*	
H10B	0.0998	−0.0105	0.0843	0.100*	
C11	0.3164 (11)	0.0675 (7)	0.0745 (5)	0.068 (3)	
H11A	0.3906	0.0087	0.0906	0.081*	
C12	0.2905 (13)	0.0667 (7)	−0.0031 (5)	0.079 (3)	
H12A	0.3957	0.0775	−0.0187	0.119*	
H12B	0.2148	0.1221	−0.0204	0.119*	
H12C	0.2446	0.0001	−0.0196	0.119*	
Cl1	0.3262 (6)	0.7906 (3)	0.19673 (17)	0.1303 (14)	
Cl2	0.3231 (4)	0.7842 (2)	0.03045 (15)	0.0970 (9)	
Cl3	0.0105 (4)	0.6753 (4)	0.0955 (2)	0.1479 (18)	
Cl4	0.2771 (8)	0.5277 (3)	0.19805 (19)	0.192 (3)	
Cl5	0.5929 (5)	0.6406 (5)	0.1308 (3)	0.199 (3)	
Cl6	0.2773 (6)	0.5279 (2)	0.02829 (18)	0.1324 (15)	
N1	0.7664 (10)	0.4296 (6)	0.2450 (5)	0.075 (2)	
H1B	0.8162	0.4831	0.2658	0.091*	
N2	0.3972 (9)	0.1656 (5)	0.1039 (4)	0.0623 (19)	
O1	0.6169 (8)	0.0811 (5)	0.1647 (4)	0.082 (2)	
Sb1	0.30206 (7)	0.65548 (4)	0.11435 (3)	0.0571 (2)	

### Atomic displacement parameters (Å^2^)

**Table:**

	*U*^11^	*U*^22^	*U*^33^	*U*^12^	*U*^13^	*U*^23^
C1	0.122 (9)	0.096 (8)	0.059 (6)	−0.040 (7)	0.024 (6)	−0.011 (6)
C2	0.165 (13)	0.102 (9)	0.048 (6)	−0.028 (9)	−0.004 (7)	−0.014 (6)
C3	0.089 (8)	0.075 (7)	0.110 (9)	−0.023 (6)	0.036 (7)	−0.017 (6)
C4	0.105 (8)	0.055 (6)	0.074 (6)	−0.022 (5)	0.035 (6)	−0.021 (5)
C5	0.047 (4)	0.050 (4)	0.040 (4)	−0.001 (3)	−0.008 (3)	0.004 (3)
C6	0.056 (5)	0.044 (4)	0.059 (5)	0.005 (4)	−0.010 (4)	0.005 (4)
C7	0.048 (5)	0.036 (4)	0.110 (7)	0.007 (4)	−0.008 (5)	0.001 (4)
C8	0.095 (9)	0.093 (8)	0.091 (8)	0.013 (7)	0.003 (6)	−0.014 (6)
C9	0.083 (8)	0.107 (10)	0.121 (10)	0.009 (7)	0.030 (7)	0.004 (8)
C10	0.094 (8)	0.078 (7)	0.076 (7)	−0.011 (6)	0.003 (6)	−0.005 (5)
C11	0.068 (6)	0.046 (5)	0.080 (6)	−0.011 (4)	−0.015 (5)	−0.006 (4)
C12	0.082 (7)	0.059 (6)	0.093 (8)	−0.001 (5)	0.004 (6)	−0.015 (5)
Cl1	0.184 (4)	0.121 (3)	0.083 (2)	−0.041 (3)	0.011 (2)	−0.042 (2)
Cl2	0.147 (3)	0.0595 (15)	0.0860 (19)	−0.0035 (16)	0.0231 (18)	0.0139 (13)
Cl3	0.0633 (18)	0.239 (5)	0.145 (3)	−0.026 (2)	0.0295 (19)	−0.056 (3)
Cl4	0.368 (8)	0.126 (3)	0.080 (2)	−0.056 (4)	0.026 (3)	0.043 (2)
Cl5	0.076 (2)	0.262 (6)	0.243 (6)	0.065 (3)	−0.019 (3)	0.038 (5)
Cl6	0.251 (5)	0.0584 (17)	0.102 (2)	−0.001 (2)	0.069 (3)	−0.0125 (16)
N1	0.069 (5)	0.056 (5)	0.091 (6)	−0.011 (4)	−0.020 (5)	−0.017 (4)
N2	0.056 (4)	0.035 (3)	0.087 (5)	0.000 (3)	−0.018 (4)	−0.002 (3)
O1	0.086 (5)	0.050 (4)	0.095 (5)	0.009 (3)	−0.032 (4)	0.012 (3)
Sb1	0.0608 (4)	0.0543 (4)	0.0552 (4)	−0.0006 (3)	0.0061 (2)	0.0038 (3)

### Geometric parameters (Å, °)

**Table:**

C1—C5	1.347 (12)	C9—C10	1.557 (15)
C1—C2	1.364 (15)	C9—H9A	0.9700
C1—H1A	0.9300	C9—H9B	0.9700
C2—N1	1.291 (15)	C10—C11	1.509 (14)
C2—H2A	0.9300	C10—H10A	0.9700
C3—N1	1.295 (13)	C10—H10B	0.9700
C3—C4	1.367 (13)	C11—N2	1.481 (10)
C3—H3A	0.9300	C11—C12	1.508 (13)
C4—C5	1.355 (11)	C11—H11A	0.9800
C4—H4A	0.9300	C12—H12A	0.9600
C5—C6	1.514 (10)	C12—H12B	0.9600
C6—O1	1.229 (9)	C12—H12C	0.9600
C6—N2	1.309 (10)	Cl1—Sb1	2.347 (3)
C7—C8	1.455 (14)	Cl2—Sb1	2.348 (3)
C7—N2	1.470 (9)	Cl3—Sb1	2.348 (3)
C7—H7A	0.9700	Cl4—Sb1	2.343 (3)
C7—H7B	0.9700	Cl5—Sb1	2.336 (4)
C8—C9	1.560 (15)	Cl6—Sb1	2.330 (3)
C8—H8A	0.9700	N1—H1B	0.8600
C8—H8B	0.9700		
			
C5—C1—C2	120.7 (10)	C9—C10—H10A	109.8
C5—C1—H1A	119.6	C11—C10—H10B	109.8
C2—C1—H1A	119.6	C9—C10—H10B	109.8
N1—C2—C1	119.6 (10)	H10A—C10—H10B	108.3
N1—C2—H2A	120.2	N2—C11—C12	112.6 (8)
C1—C2—H2A	120.2	N2—C11—C10	108.1 (8)
N1—C3—C4	120.6 (10)	C12—C11—C10	113.0 (8)
N1—C3—H3A	119.7	N2—C11—H11A	107.6
C4—C3—H3A	119.7	C12—C11—H11A	107.6
C5—C4—C3	119.3 (9)	C10—C11—H11A	107.6
C5—C4—H4A	120.4	C11—C12—H12A	109.5
C3—C4—H4A	120.4	C11—C12—H12B	109.5
C1—C5—C4	117.8 (8)	H12A—C12—H12B	109.5
C1—C5—C6	119.6 (8)	C11—C12—H12C	109.5
C4—C5—C6	122.2 (7)	H12A—C12—H12C	109.5
O1—C6—N2	125.3 (7)	H12B—C12—H12C	109.5
O1—C6—C5	115.6 (7)	C2—N1—C3	122.1 (9)
N2—C6—C5	119.0 (6)	C2—N1—H1B	119.0
C8—C7—N2	110.4 (8)	C3—N1—H1B	119.0
C8—C7—H7A	109.6	C6—N2—C7	125.2 (7)
N2—C7—H7A	109.6	C6—N2—C11	120.4 (6)
C8—C7—H7B	109.6	C7—N2—C11	114.0 (6)
N2—C7—H7B	109.6	Cl6—Sb1—Cl5	90.8 (2)
H7A—C7—H7B	108.1	Cl6—Sb1—Cl4	91.26 (14)
C7—C8—C9	112.0 (9)	Cl5—Sb1—Cl4	92.5 (2)
C7—C8—H8A	109.2	Cl6—Sb1—Cl1	177.11 (13)
C9—C8—H8A	109.2	Cl5—Sb1—Cl1	89.5 (2)
C7—C8—H8B	109.2	Cl4—Sb1—Cl1	91.60 (16)
C9—C8—H8B	109.2	Cl6—Sb1—Cl3	89.46 (16)
H8A—C8—H8B	107.9	Cl5—Sb1—Cl3	178.1 (2)
C10—C9—C8	109.8 (10)	Cl4—Sb1—Cl3	89.4 (2)
C10—C9—H9A	109.7	Cl1—Sb1—Cl3	90.17 (15)
C8—C9—H9A	109.7	Cl6—Sb1—Cl2	88.89 (11)
C10—C9—H9B	109.7	Cl5—Sb1—Cl2	88.32 (18)
C8—C9—H9B	109.7	Cl4—Sb1—Cl2	179.19 (19)
H9A—C9—H9B	108.2	Cl1—Sb1—Cl2	88.24 (13)
C11—C10—C9	109.3 (9)	Cl3—Sb1—Cl2	89.84 (16)
C11—C10—H10A	109.8		
			
C5—C1—C2—N1	−0.4 (11)	C9—C10—C11—C12	−66.8 (11)
N1—C3—C4—C5	−0.3 (14)	C1—C2—N1—C3	0.1 (13)
C2—C1—C5—C4	0.3 (13)	C4—C3—N1—C2	0.2 (15)
C2—C1—C5—C6	173.5 (7)	O1—C6—N2—C7	176.7 (9)
C3—C4—C5—C1	0.1 (14)	C5—C6—N2—C7	−4.7 (14)
C3—C4—C5—C6	−173.0 (8)	O1—C6—N2—C11	4.7 (15)
C1—C5—C6—O1	−77.7 (11)	C5—C6—N2—C11	−176.7 (8)
C4—C5—C6—O1	95.2 (11)	C8—C7—N2—C6	−112.8 (10)
C1—C5—C6—N2	103.5 (10)	C8—C7—N2—C11	59.6 (11)
C4—C5—C6—N2	−83.6 (12)	C12—C11—N2—C6	−123.4 (10)
N2—C7—C8—C9	−53.9 (12)	C10—C11—N2—C6	111.1 (10)
C7—C8—C9—C10	53.4 (13)	C12—C11—N2—C7	63.8 (11)
C8—C9—C10—C11	−55.2 (12)	C10—C11—N2—C7	−61.8 (11)
C9—C10—C11—N2	58.5 (10)		

### Hydrogen-bond geometry (Å, °)

**Table:**

*D*—H···*A*	*D*—H	H···*A*	*D*···*A*	*D*—H···*A*
N1—H1B···O1^i^	0.86	1.87	2.689 (9)	159

Symmetry codes: (i) −*x*+3/2, *y*+1/2, −*z*+1/2.
